# Limited effects of dysfunctional macroautophagy on the accumulation of extracellularly derived α-synuclein in oligodendroglia: implications for MSA pathogenesis

**DOI:** 10.1186/s12868-018-0431-2

**Published:** 2018-05-21

**Authors:** Lisa Fellner, Edith Buchinger, Dominik Brueck, Regina Irschick, Gregor K. Wenning, Nadia Stefanova

**Affiliations:** 1 Department of Neurology, Medical University of Innsbruck, Innrain 66, G2, 6020 Innsbruck, Austria; 2 Department of Anatomy, Histology and Embryology, Medical University of Innsbruck, Anichstrasse 35, Innsbruck, Austria

**Keywords:** Macroautophagy, Multiple system atrophy, α-Synuclein, Oligodendroglia, Glial cytoplasmic inclusions

## Abstract

**Background:**

The progressive neurodegenerative disorder multiple system atrophy (MSA) is characterized by α-synuclein-positive (oligodendro-) glial cytoplasmic inclusions (GCIs). A connection between the abnormal accumulation of α-synuclein in GCIs and disease initiation and progression has been postulated. Mechanisms involved in the formation of GCIs are unclear. Abnormal uptake of α-synuclein from extracellular space, oligodendroglial overexpression of α-synuclein, and/or dysfunctional protein degradation including macroautophagy have all been discussed. In the current study, we investigated whether dysfunctional macroautophagy aggravates accumulation of extracellular α-synuclein in the oligodendroglia.

**Results:**

We show that oligodendroglia uptake monomeric and fibrillar extracellular α-synuclein. Blocking macroautophagy through bafilomycin A1 treatment or genetic knockdown of LC3B does not consistently change the level of incorporated α-synuclein in oligodendroglia exposed to extracellular soluble/monomeric or fibrillar α-synuclein, however leads to higher oxidative stress in combination with fibrillar α-synuclein treatment. Finally, we detected no evidence for GCI-like formation resulting from dysfunctional macroautophagy in oligodendroglia using confocal microscopy.

**Conclusion:**

In summary, isolated dysfunctional macroautophagy is not sufficient to enhance abnormal accumulation of uptaken α-synuclein in vitro, but may lead to increased production of reactive oxygen species in the presence of fibrillar α-synuclein. Multiple complementary pathways are likely to contribute to GCI formation in MSA.

## Background

Multiple system atrophy (MSA) is a progressive, fatal neurodegenerative disease with unknown etiology. MSA is characterized by α-synuclein (α-syn)-positive glial cytoplasmic inclusions (GCIs) occurring predominantly in oligodendroglial cells [1]. MSA is categorized in the disease group of α-synucleinopathies together with Parkinson’s disease (PD) and dementia with Lewy bodies (DLB) which show primarily neuronal α-syn-positive inclusions (Lewy bodies, LBs). Different genetic, neuropathological and experimental studies provide evidence that α-syn plays a major role in the pathogenesis of these disorders [2–9]. Yet, phosphorylated and aggregated α-syn species are not the only constituents of GCIs, but also ubiquitin, heat shock proteins, cytoskeletal proteins and components of the autophagic pathway were identified [10–14]. The formation of GCIs in MSA and the underlying mechanisms are not elucidated to date [15]. An elevated expression and aggregation of α-syn in oligodendroglial cells is discussed in GCI development, yet contradicting reports exist regarding the expression of α-syn mRNA in oligodendroglial cells [16–19]. Incorporation of α-syn by oligodendroglia either released by dying neurons into the extracellular space or by cell-to-cell propagation represents an alternative mechanism of GCI formation [20–22]. The uptake of α-syn via endocytosis by oligodendroglial cells has been reported in different studies [13, 22–26], but α-syn uptake by oligodendrocytes has not been immediately linked to the formation of GCIs. Furthermore, the degradation of monomeric/soluble α-syn (sol α-syn) by the ubiquitin-proteasome system (UPS) and autophagy has been demonstrated [27–29], whereas autophagy seems to be the favored pathway for the degradation of misfolded α-syn species [30]. Therefore, pathological accumulation of α-syn in the cytoplasm of oligodendroglia might be explained by a primary oligodendroglial injury [31] such as deficits in the cellular protein degradation mechanisms [11, 32, 33].

Three different forms of autophagy are described: macro-, microautophagy and chaperone-mediated autophagy (CMA). Macroautophagy and CMA have been shown to be the most relevant autophagy mechanisms involved in the degradation of α-syn [34]. Macroautophagy (commonly called autophagy) is important for the bulk degradation of cytoplasmic proteins or organelles and thereby involves autophagic vacuoles (autophagosomes) that fuse with lysosomes to form autophagolysosomes where the degradation via hydrolases takes place [35]. The formation of the autophagosome is a complex process which is controlled by various evolutionary conserved ATG (AuTophaGy) genes as well as lipid kinases [36, 37]. A standard assay for macroautophagy is the measurement of autophagosome-associated LC3B protein levels. The processing of microtubule-associated protein 1 light chain 3B (LC3B) creates LC3B-I and after its lipidation LC3B-II develops, which is a robust marker of autophagosomes and correlates with autophagosome numbers [38, 39].

Impaired autophagy associated with deficits in the autophagosome formation has been reported in MSA [40], suggesting that this dysfunction might initiate GCI formation in oligodendroglia [41–43]. In PD brains, macroautophagy was shown to be impaired in nigral neurons [44]. In a recent study, it was found that the autophagosomal protein LC3 and the ubiquitin binding protein p62 are associated with α-syn-positive GCIs in MSA cases and furthermore, LC3 is recruited to α-syn aggregations when the proteasome is impaired in rat oligodendroglial cells [11, 45]. In a transgenic MSA mouse model, an upregulation of the LC3 protein was demonstrated compared to wild type mice strengthening the assumption of macroautophagy involvement in MSA-like α-synucleinopathy [32]. In addition, dysfunctional macroautophagy caused through mitochondrial impairment or macroautophagy inhibition resulted in the accumulation of α-syn in oligodendroglial cells in vitro respectively [24]. Furthermore, in a different study in neuronal cells a role of CMA and macroautophagy were shown in the degradation process of α-syn [29]. Recently, it was demonstrated that macroautophagy inhibition by using bafilomycin A1 (BAF), a specific inhibitor of vacuolar H + ATPase (V-ATPase) blocking the fusion of the autophagosome and the lysosome [46], reduced intracellular α-syn aggregation, but also led to an enhanced secretion of toxic α-syn oligomers by neuronal cells in vitro and in vivo thereby causing inflammation and neurotoxicity in the microenvironment [42, 47]. These studies underline the involvement of macroautophagy in the aggregation and degradation process of α-syn especially in neuronal, but also in oligodendroglial cells. Yet, the complete mechanisms of α-syn inclusion formation in MSA and the role of autophagy pathways are not elucidated to this date and have to be further investigated to create an overall picture.

In the present study, we investigated the effect of macroautophagy inhibition and exposure to extracellular recombinant α-syn species on the formation of α-syn-positive inclusions in human oligodendroglial cells. Therefore, pharmacological blocking using BAF or genetic knockdown of LC3B was analyzed in oligodendroglial cells exposed to extracellular monomeric/soluble (sol α-syn) or fibrillar α-syn (fib α-syn) species. Macroautophagy block through genetic knockdown of LC3 or BAF treatment was inefficient to increase intracellular accumulation of α-syn in oligodendrocytes exposed to extracellular α-syn. In the current study, no GCI-like formation of α-syn upon macroautophagy blocking was found in the used oligodendroglial cell culture model suggesting that multiple complementary factors are likely to contribute to GCI formation in MSA.

## Methods

### Preparation, purification and characterization of full length soluble and fibrillar α-syn

The recombinant human full length monomeric sol α-syn was prepared, purified and characterized as described previously [5, 15, 48]. Purification was performed using a histidin-tag attached to the protein. This his-tag leads to a 24 kilodalton (kDa) band when analyzed in western blot analysis. Recombinant human full length fib α-syn was generated and fibrillization was characterized using Thioflavin T (Sigma-Aldrich, St. Louis, MO, USA) as specified before [15]. Furthermore, endotoxin concentration in the α-syn preparations was determined by using the kinetic chromogenic limulus amoebocyte lysate (LAL) endpoint assay by Hyglos GmbH, Bernried, Germany reaching an endotoxin amount under 1 EU/mg in the stock solution as described previously [15].

### Cell culture

As part of the current study, a primary murine oligodendroglial cell culture was established using newborn wild type (C57BL/6) mouse cortices (days 1–3). According to national regulations of the Austrian Animal experimentation law (TVG 2012), no ethics approval is necessary for the preparation of primary cell culture. Newborn mice were sacrificed and whole cortices prepared. Meninges were removed and mixed glial cultures were obtained as described before and were kept in culture for 2 weeks [15]. The mixed glial cell culture was shaken consecutively to generate a primary oligodendrocyte precursor cell (OPC) culture by separating OPCs from the underlying astroglial layer. Thereby, murine mixed glial cultures were pre-shaken for 1 h at 180 rpm on a horizontal orbital shaker at 37 °C to remove microglial cells. Medium with microglia was discarded and 10 ml of complete mixed glial medium was added. Then the flasks were shaken at 180 rpm overnight (18–20 h) at 37 °C in a humid atmosphere with 5% CO_2_. To separate OPCs from microglia and remaining astroglia, cells were plated onto an untreated petri dish (BD Falcon, BD Biosciences, BD, San Jose, CA, USA) for 40 min to induce adherence of microglia and astroglia to the plastic. OPCs remaining in the medium were centrifuged and replated onto poly-d-lysine (PDL, 20 µg/mL, Gibco, Life Technologies, San Diego, CA, USA) coated 96-well plates (4 × 10^4^ cells per well, TPP, Trasadingen, Switzerland) in OPC-conditioned medium containing basic fibroblast growth factor (bFGF, 10 ng/mL, Life Technologies) and platelet-derived growth factor AA (PDGF-AA, 10 ng/mL, Life Technologies) at 37 °C in a humid atmosphere with 5% CO_2_ as described previously [49]. OPCs were differentiated after 5–6 days in culture using a special maturation mix including triiodothyronine (TIT, 15 nM, Sigma-Aldrich), N-acetyl-l-cysteine (NAC, 1×, Sigma-Aldrich) and ciliary neurotrophic factor (CNTF, 10 ng/mL, PeproTech, Hamburg, Germany) as specified by Chen et al. [49].

The human oligodendroglioma cell line MO 3.13 (Cedarlane, Ontario, Canada) was kept in T75 flasks (TPP) in Dulbecco’s modified Eagle’s medium (DMEM, 4 g/L Glucose, Gibco, Life Technologies) supplemented with 10% fetal calf serum (FCS, Gibco, Life Technologies) and 2 mM Glutamine (Gibco, Life Technologies). Cells were passaged twice a week and kept at 37 °C in a humid atmosphere with 5% CO_2_.

### α-syn uptake by primary murine oligodendroglial cells and MO 3.13 oligodendroglial cells

Primary murine oligodendroglia were plated into  PDL coated 96-well plates (4 × 10^4^ cells per well) and MO 3.13 oligodendroglial cells were plated into 96-well plates (5 × 10^3^ cells per well). After 24 h, the cells were treated with  18 µg/mL recombinant sol and fib α-syn for 1 or 24 h at 37 °C. Cells were washed with PBS, and then fixed with 4% paraformaldehyde (PFA, Sigma-Aldrich) and immunocytochemistry was conducted. The uptake of sol and fib α-syn was analyzed using a DMI 4000B Leica inverse microscope provided with Leica application software and Digital FireWire Color Camera DFC300 FX (Leica Microsystems). All measurements were repeated in at least 4 separate biological replicates and mean values (± SEM) were determined.

### Induction of macroautophagy dysfunction by pharmacological blocking

Autophagy dysfunction was generated by the addition of BAF (50 nM, Sigma-Aldrich) to the medium, thereby blocking the fusion of the autophagosome with the lysosome. MO 3.13 were plated into 96-well plates (5 × 10^3^ cells per well) for the performance of immunocytochemistry or into 6-well plates (26 × 10^4^ cells per well) for the conduction of western blot. 50 nM BAF was applied for 30 min to induce autophagy dysfunction in MO 3.13 cells. Subsequently, 18 µg/mL sol or fib α-syn was added. Cells were either fixed with 4% PFA and immunocytochemistry was accomplished or lysates were generated.

### Sure silencing shRNA plasmids and transfection

To achieve a knockdown of macroautophagy via LC3B silencing using RNA interference in MO 3.13 oligodendroglia, the SureSilencing shRNA plasmids ligated to green fluorescent protein (GFP) were generated according to the manufacturer’s instructions (Qiagen, Hilden, Germany). LC3B shRNA plasmid sequence: GCAGCTTCCTGTTCTGGATAA; scrambled shRNA plasmid sequence: ggaatctcattcgatgcatac.

FuGENE Transfection Reagent (Promega, Mannheim, Germany) was applied to perform transfections of MO 3.13 cell cultures as recommended by the manufacturer. Cells were plated 1 day prior to transfection so that they were approximately 80% confluent. 72 h (for Western blot analyses) and 24 h (for confocal microscopy) after transfection with the SureSilencing shRNA plasmids the medium was changed and the cells were treated with  18 µg/mL recombinant human sol α-syn or fib α-syn for 24 h. α-Syn uptake or/and inclusion formation was determined by immunostaining and immunoblotting.

### Immunofluorescence

The following primary antibodies were used in this study: rat anti-human α-syn (aa 116-131 human α-syn, 1:500, Enzo Life Sciences, Loerrach, Germany), rabbit anti-human ubiquitin (1:200, Abcam, Cambridge, UK) and rabbit-anti mouse PDGF receptor α (PDGFRα, 1:200, Abcam). Cell culture medium was removed and cells were washed twice with phosphate buffered saline (PBS) prior to fixation with 4% PFA for 20 min at room temperature (RT). Subsequently, cells were blocked in 0.3% Triton-X100, 1% bovine serum albumin (Sigma-Aldrich) and 5% normal goat serum (Gibco, Life Technologies) in PBS for 1 h followed by incubation with primary antibody overnight at 4 °C. Secondary antibodies for immunofluorescence were added for 1 h at RT, including Alexa 488- or Alexa 594-conjugated anti-rat and anti-rabbit IgG (1:500, Jackson Immunoresearch Laboratories, West Grove, PA, USA). Nuclear staining of fixed cells was accomplished using 4′,6-diamidino-2-phenylindole dihydrochloride (DAPI, 1:20,000 Sigma-Aldrich). Membrane staining was achieved by detection of glycoproteins using FITC-conjugated lectin from Triticum vulgaris (wheat germ agglutinin, WGA, 1:1000, Sigma-Aldrich). Cells were visualized using a DMI 4000B Leica inverse microscope and Application Suite V3.1 and Digital Fire Wire Color Camera DFC300 FX by Leica or by confocal microscopy. For confocal microscopy stained oligodendroglial cells were mounted on slides and coverslipped.

### Confocal microscopy

Confocal microscopy was conducted at a Leica TCS SP5 laser scanning microscope (Leica Microsystems, Wetzlar, Germany) using a HCX PL APO 63x glycerol immersion objective (N.A. 1.3) and a pinhole of 1 AU. Three-dimensional stacks were acquired according to the Nyquist criterion to allow deconvolution. Image deconvolution was performed using Huygens professional software version 4.1.1 (SVI Scientific Volume Imaging, Hilversum, The Netherlands). Images were processed using the Huygens Object Analyzer Advanced and cells were reconstructed three-dimensionally with the Huygens MIP renderer.

### Measurement of ROS

Intracellular superoxide radical generation assessment was accomplished using nitroblue tetrazolium chloride (NBT, Roche Applied Sciences, Basel, Switzerland). Thereby, the formation of a dark blue formazan deposit resulting from superoxide-mediated reduction of NBT can be examined in cell culture visualizing cells positive for ROS production as described previously [15]. Briefly, MO 3.13 oligodendroglial cells were plated in 96-well plates and treated as described before. Cells were treated with BAF and recombinant α-syn. Untreated cells were used as controls. Subsequently, 1 mg/mL NBT was added at 37 °C for 30 min. Cells were washed with PBS and fixed with 4% PFA at RT. The number of ROS-positive cells per well was analyzed by using a DMI 4000B Leica inverse microscope. All measurements were repeated in at least 4 separate biological replicates and mean values (± SEM) were determined.

### SDS-PAGE and western blot

Lysates of treated MO 3.13 oligodendroglia were generated. Cells were washed with PBS once, and lysed in protein lysis buffer containing 1% NP-40, 150 mM NaCl, 50 mM HEPES, 0.8 mM MgCl_2_, 5 mM EGTA protease inhibitor cocktail (Roche Applied Sciences). Lysates were centrifuged for 15 min at 4 °C and the supernatant was stored at − 80 °C. Protein content of the cell lysates was determined using the BCA protein assay (Sigma-Aldrich). Electrophoresis of cell lysates was accomplished using NuPAGE 10% Bis–Tris gels (Novex Life Technologies) for protein separation. Proteins were electrotransferred to a nitrocellulose membrane (GE Healthcare Bio-Sciences AB, Uppsala, Sweden) and after blocking with 2% milk powder in PBS containing 0.1% Tween-20 (PBS-T), the blots, were incubated with different primary antibodies overnight, including purified monoclonal AS antibody (aa 15-123, 1:1000, BD Transduction Laboratories, San Jose, CA, USA), monoclonal actin antibody (housekeeper, 1:10,000, BD Transduction Laboratories), polyclonal ubiquitin antibody (1:2000, Abcam) and a polyclonal LC3B antibody (1:1000, Cell Signaling, Leiden, The Netherlands). Blots were further incubated with horseradish peroxidase linked anti-mouse or anti-rabbit IgG (1:10,000, GE Healthcare) and incubated for another 2 min with Western Bright enhanced chemiluminescence (ECL, Advansta, Menlo Park, CA, USA). The blots were developed using the imaging system Fusion Fx 7 for Western blot and gel imaging, quantification was performed using the FUSION CAPT V16.09b Software (Vilber Lourmat, Marne La Vallée, France). All measurements were repeated in at least 4 separate biological replicates and mean values (± SEM) were determined.

### Statistical analysis

All statistical analyses were carried out using Graph-Pad Prism 5 (Graphpad Software, San Diego, CA, USA) and the results were presented as the mean ± S.E.M. Two-way analysis of variance with post hoc Bonferroni test for the analysis of two independent factors was applied. A *p* value < 0.05 was considered statistically significant.

## Results

### Primary murine oligodendrocytes and human oligodendroglial cell line incorporate extracellular α-syn

**Fig. 1:**
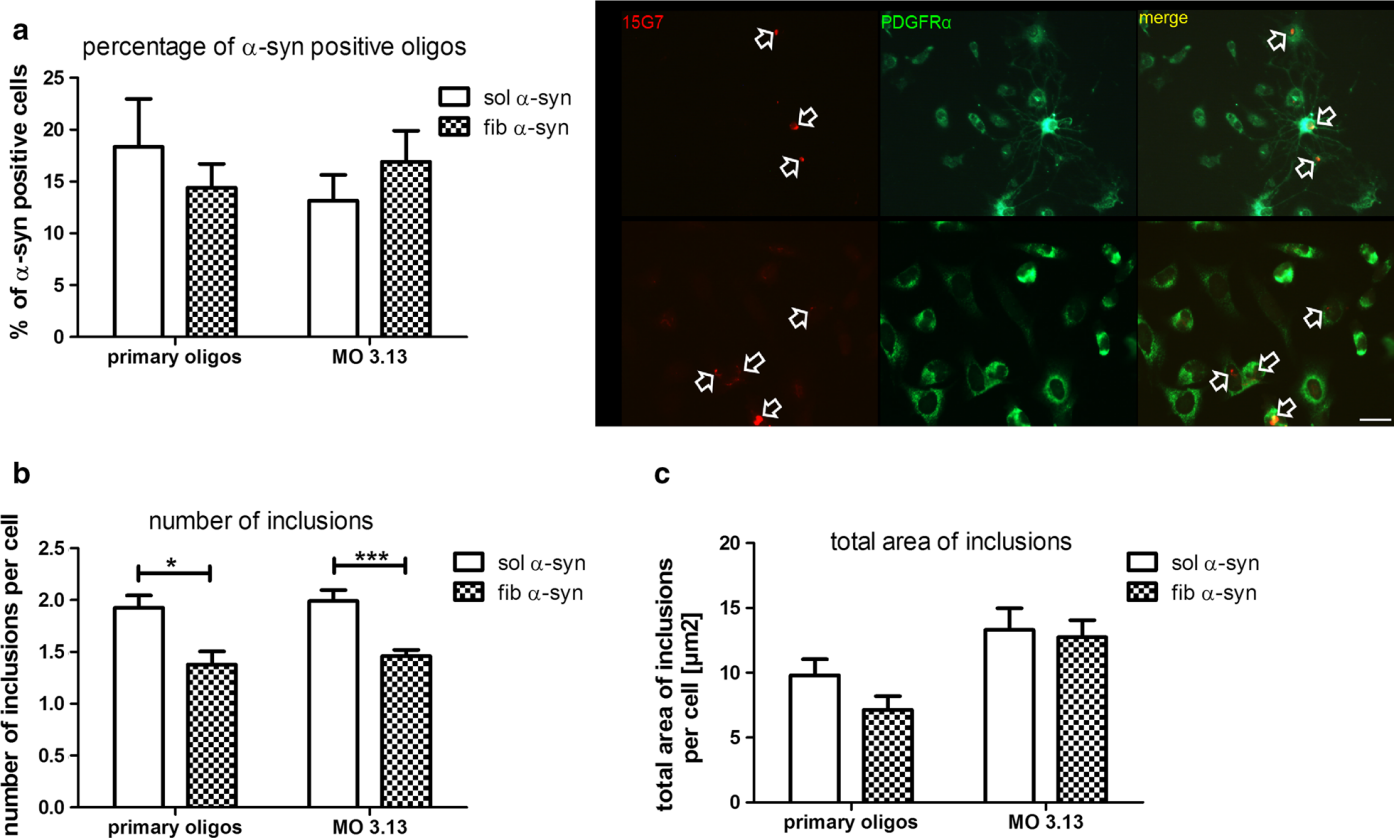
Incorporation of α-syn by murine primary oligodendroglia as compared to the human oligodendroglioma cell line MO 3.13. Murine primary oligodendroglial cells (primary oligos) and the human oligodendroglial cell line MO 3.13 were treated with 18 µg/mL sol and fib α-syn for 24 h. Immunocytochemistry for α-syn (15G7, red) and PDGFRα (green) were performed and the evaluation was done by an unbiased investigator. The number of α-syn-positive oligodendroglial cells was evaluated and the percentage of α-syn-positive cells was calculated showing no difference between primary and MO 3.13 oligodendroglia. Arrows showing inclusions positively stained for 15G7 in the cytoplasm of primary and MO 3.13 oligodendroglial cells (**a**). The number of α-syn-positive inclusions per cell was counted revealing fewer inclusions per cell upon fib α-syn compared to sol α-syn exposure in both cell types (**b**). Moreover, the total area of inclusions per cell (µm^2^) was measured indicating a slight increase of the area in MO 3.13 compared to primary oligodendroglia; however, the difference did not reach statistical significance (**c**). Two-way analysis of variance with post hoc Bonferroni test was applied. Data are presented as mean ± SEM *p < 0.05; ***p < 0.001. Scale bar 20 µm. N number equals 4

Previous in vitro studies already suggested the incorporation of α-syn by oligodendroglial cell lines and primary oligodendroglial cells [22, 25, 26]. To determine whether recombinant sol and fib α-syn incorporation from the extracellular space is comparable in primary murine oligodendroglia and human MO 3.13 oligodendroglial cell line, we analyzed different parameters including the percentage of cells with α-syn inclusions (Fig. 1a), the number of inclusions per cell (Fig. 1b) and the total area of inclusion per cell in µm^2^ (Fig. 1c). 24 h after incubation with both α-syn forms, small incorporations of α-syn were found in around 15–20% of the analyzed primary and MO 3.13 oligodendroglia. No differences were detected regarding the cell type and the α-syn species (Fig. 1a). Furthermore, the number of inclusions per cell was similar in primary and MO 3.13 oligodendroglia. Yet, fib α-syn treatment presented with a significant lower number of inclusions per cell compared to sol α-syn in both cell types (Fig. 1b). Furthermore, we did not observe a statistically significant difference measuring the total area of the inclusions per cell for both α-syn types in primary oligodendroglial cells compared to the oligodendroglial cell line MO 3.13, however a tendency to smaller inclusions in primary oligodendroglial cells was found (Fig. 1c).

### BAF-induced dysfunctional macroautophagy in oligodendroglial cells does not trigger GCI-like formation from extracellularly uptaken α-syn

**Fig. 2:**
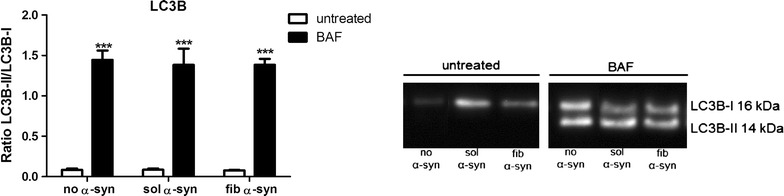
Confirmation of the block of macroautophagy by treatment with BAF in oligodendroglial cells. Cell lysates were analyzed by Western blotting and the band intensities for LC3B-II were normalized to LC3B-I levels. Increased LC3B-II levels indicate the successful block of the fusion of the autophagosome with the lysosome. Oligodendroglial cells untreated or treated with 18 µg/mL sol or fib α-syn for 24 h showed a low ratio of LC3B-II/LC3B-I, whereas BAF-treated oligodendroglia revealed significantly higher levels of the LC3B-II/LC3B-I ratio independent of the α-syn treatment as compared to oligodendroglia not treated with BAF. Two-way analysis of variance with post hoc Bonferroni test was applied. Data are presented as mean ± SEM. ***p < 0.001. N number equals 4

**Fig. 3:**
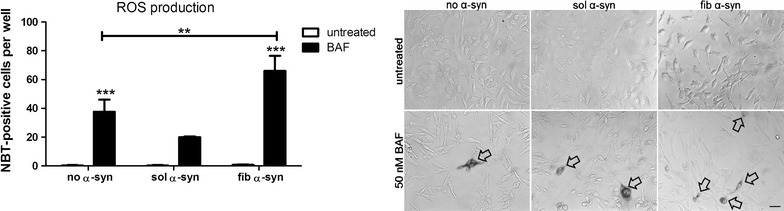
Blocking macroautophagy with BAF in combination with α-syn treatment results in increased ROS production in oligodendroglial cells. Following treatment, cells were stained for ROS by using nitroblue tetrazolium chloride (NBT) resulting in a blue precipitate in the presence of ROS. We found that treatment with BAF significantly enhances the number of NBT-positive oligodendroglial cells compared to untreated cells. Furthermore, the additional treatment with extracellular fib α-syn for 24 h revealed a significant increase of NBT-positive cells compared to cells treated with BAF only and cells not treated with BAF. However, treatment with sol α-syn did not induce a significant increase of ROS production. Arrows pointing out oligodendroglial cells positively stained for NBT. Two-way analysis of variance with post hoc Bonferroni test was applied. Data are presented as mean ± SEM. **p < 0.01; ***p < 0.001. Scale bar 20 µm. N number equals 4

To evaluate the effect of blocking the fusion of the autophagosome and the lysosome on GCI-like formation in oligodendroglia, cells were treated with the pharmacological autophagy inhibitor BAF followed by exposure to sol or fib α-syn. As BAF blocks LC3B-II degradation, successful macroautophagy inhibition can be identified by increased LC3B-II levels [50, 51]. Western blot analysis of BAF-treated oligodendroglia revealed a significant increase of LC3B-II levels in our experiments irrespective of an additional sol or fib α-syn treatment. Comparing untreated cells and sole addition of sol or fib α-syn, no change in the levels of LC3B-II was found indicative of a normal autophagic flux (Fig. 2). Interestingly, we found that blocking of macroautophagy with BAF induces oxidative stress as measured by the formation of ROS (Fig. 3). Treating oligodendroglia with the two types of extracellularly added α-syn only did not induce oxidative stress compared to cells treated with BAF and α-syn as quantified ROS levels reveal. The addition of extracellular recombinant fib α-syn, however not with sol α-syn, showed a significant increase of NBT-positive cells in BAF-treated oligodendroglia compared to untreated cells (Fig. 3).

**Fig. 4:**
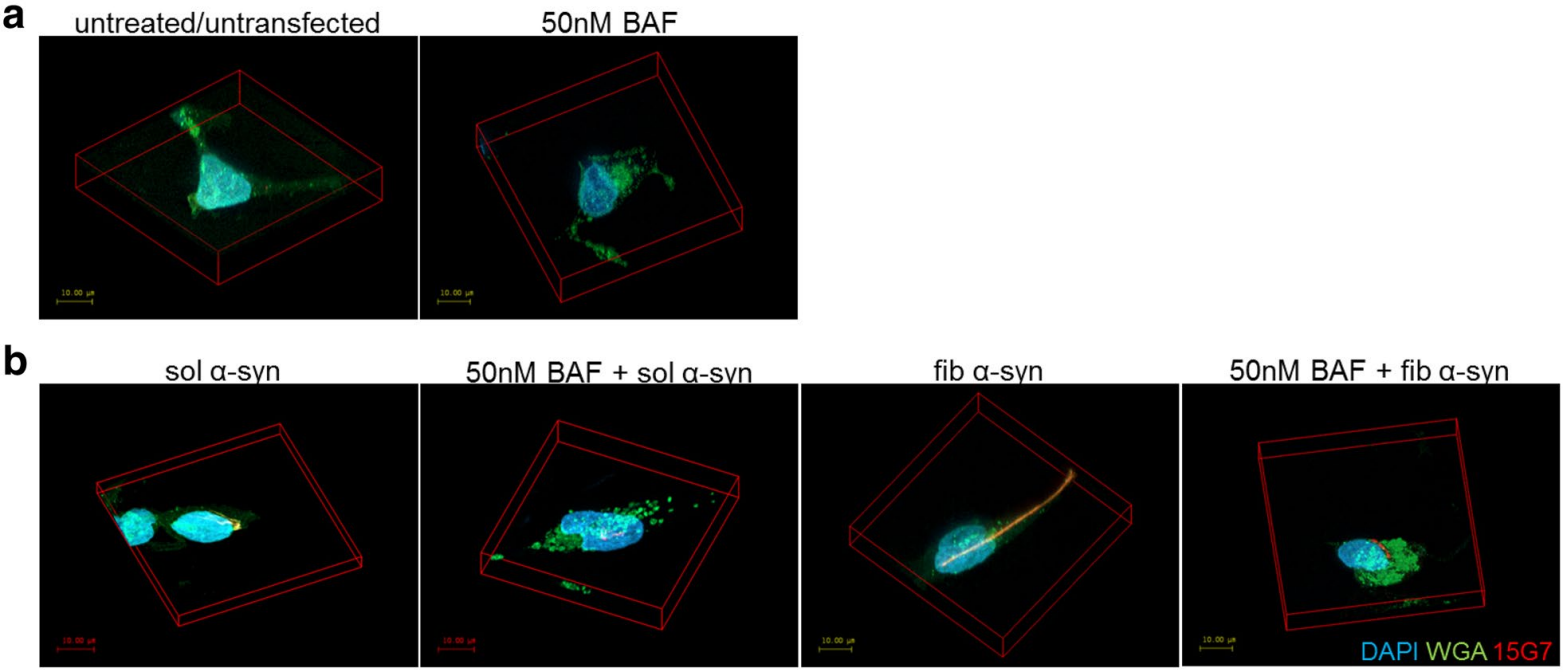
Morphological analysis of α-syn-positive inclusions upon macroautophagy blocking treatment in oligodendroglial cells. Confocal microscopy was performed to analyze the incorporation and inclusion formation of sol and fib α-syn in oligodendroglial cells challenged with BAF for 24 h. Immunocytochemistry was accomplished following fixation, labelling human α-syn (15G7, red), the membrane using wheat germ agglutinin (WGA, green) and the nucleus using DAPI (blue). Treatment with BAF induced a more pronounced vesicle formation as can be seen by the membrane staining with WGA (**a**). The addition of recombinant sol and fib α-syn for 24 h to the medium induced the incorporation of α-syn by oligodendroglial cells. Yet, simultaneous macroautophagy blocking did not induce an increased amount of α-syn incorporated by these oligodendroglial cells. No GCI-like formation was observed upon macroautophagy block and treatment with extracellularly added sol or fib α-syn. In some cells translocation of α-syn to the nucleus was observed as shown in picture fib α-syn. Moreover, BAF treatment induced an enhanced number of vesicles in the cytoplasm (WGA staining) irrespective of α-syn in the cytoplasm of the cells (**b**). Scale bar 10 µm

As shown by other groups, oligodendroglial cells are capable to uptake α-syn from the extracellular space [22, 25, 26]. We confirm here the incorporation of extracellularly added sol and fib α-syn in oligodendroglial cells by confocal microscopy (Fig. 4). BAF treatment induced vesicle formation in the cytoplasm as seen by WGA-stained membranes (Fig. 4a). However, we were not able to generate GCI-like aggregates by blocking the fusion of the autophagosome with the lysosome using 50 nM BAF irrespective of the used extracellular α-syn form after 24 h. Interestingly, sol and fib α-syn were not only distributed in the cytoplasm but also a translocation to the nucleus of α-syn was found (Fig. 4b). BAF induced the generation of vesicles in the cytoplasm as stained with the lectin compound WGA linked to FITC suggesting inhibited macroautophagy (Fig. 4). Yet, α-syn was not found to co-localize with the WGA-positive vesicles (Fig. 4b).

**Fig. 5:**
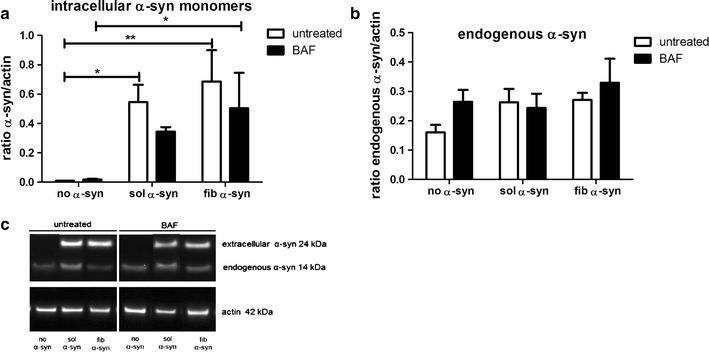
Incorporation of extracellular monomeric sol and fib α-syn species and inclusion formation in oligodendroglial cells upon macroautophagy blocking with BAF. Intracellular amounts of uptaken α-syn monomers and endogenous α-syn were measured in oligodendroglial cells challenged with extracellular sol or fib α-syn for 24 h combined with macroautophagy blocking for 24 h using Western blot analysis. α-syn levels were normalized to actin. Addition of sol and fib α-syn led to a significantly increased amount of α-syn in oligodendroglial cells (24 kDa band due to his-tag). Moreover, an increased amount of α-syn was found in oligodendroglia treated with BAF compared to cells not treated with α-syn, however a significant change was detected only after fib α-syn exposure. Comparing untreated cells/cells treated with BAF and α-syn a not significant decrease regarding the amounts of α-syn was measured (**a**,** c**). Levels of endogenously expressed α-syn (14 kDa band, no his-tag) remained the same regarding all relevant treatments (**b**,** c**). Two-way analysis of variance with post hoc Bonferroni test was applied. Data are presented as mean ± SEM. *p < 0.05; **p < 0.01. N number equals 4

Western blot analysis of intracellular α-syn supported these morphological observations. Oligodendroglial cells incorporated a significant amount of extracellularly added sol or fib α-syn. Yet, the inhibition of macroautophagy through BAF had no significant effect on the amounts of intracellular α-syn and did not induce a pathological aggregation of α-syn in oligodendroglial cells as no oligomers or fibrils were detected (Fig. 5a). Moreover, the oligodendroglial cells used for these experiments, express a very low amount of endogenous α-syn. However, the blocking of macroautophagy with the pharmacological blocker BAF did not induce an accumulation of the endogenously expressed α-syn. Discrimination between endogenous and extracellular α-syn was possible as the extracellularly added α-syn had due to an attached his-tag an increased size (24 kDa) compared to endogenously expressed α-syn (14 kDa) (Fig. 5).

**Fig. 6:**
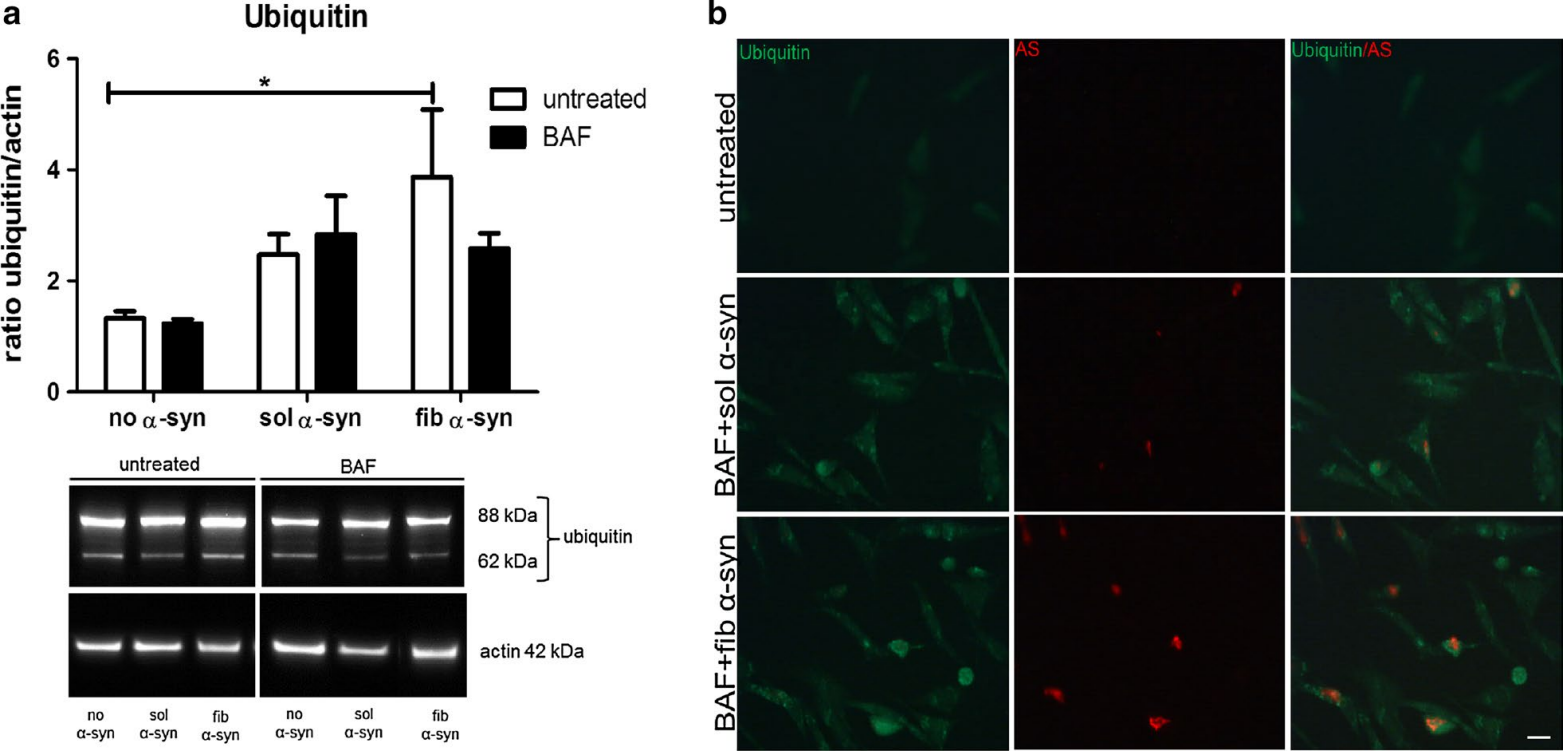
Inhibited macroautophagy is not associated with increased ubiquitin levels in oligodendroglia treated with α-syn. Intracellular amounts of ubiquitin were measured in oligodendroglial cells exposed to extracellular sol or fib α-syn using Western blot analysis. Ubiquitin levels were normalized to actin levels (**a**). Furthermore, co-localization of ubiquitin and α-syn was analyzed using immunocytochemistry, labelling human α-syn (15G7) red and ubiquitin green (**b**). A slight increase of ubiquitin levels was measured upon treatment with sol and fib α-syn and BAF compared to untreated oligodendroglial cells. However, only fib α-syn treatment alone induced a significant increase of ubiquitin levels (**a**). Furthermore, no co-localization of ubiquitin staining (green) and α-syn (red) was found upon treatment with BAF and sol or fib α-syn. Scale bar 20 µm (**b**). Two-way analysis of variance with post hoc Bonferroni test was applied. Data are presented as mean ± SEM. *p < 0.05. N number equals 4

Furthermore, we analyzed the levels of another protein most common in GCIs, namely ubiquitin [52]. As ubiquitination is abundant in GCIs, higher levels of ubiquitin might indicated GCI-like formation in oligodendroglial cells. In this cell culture model a slight increase of ubiquitin levels upon treatment with BAF and extracellularly added α-syn or with extracellularly added α-syn only was detected. Yet, a significant increase of ubiquitin levels was achieved only by challenging oligodendroglial cells with fib α-syn compared to untreated cells. Macroautophagy blocking using BAF followed by α-syn treatment did not increase ubiquitin levels after 24 h of treatment (Fig. 6a). Furthermore, no co-localization of α-syn and ubiquitin was found upon treatment with BAF and sol or fib α-syn as investigated using fluorescence microscopy (Fig. 6b).

### Genetic knockdown of LC3B in oligodendroglial cells does not induce the formation of GCI-like aggregates from extracellularly uptaken α-syn

**Fig. 7:**
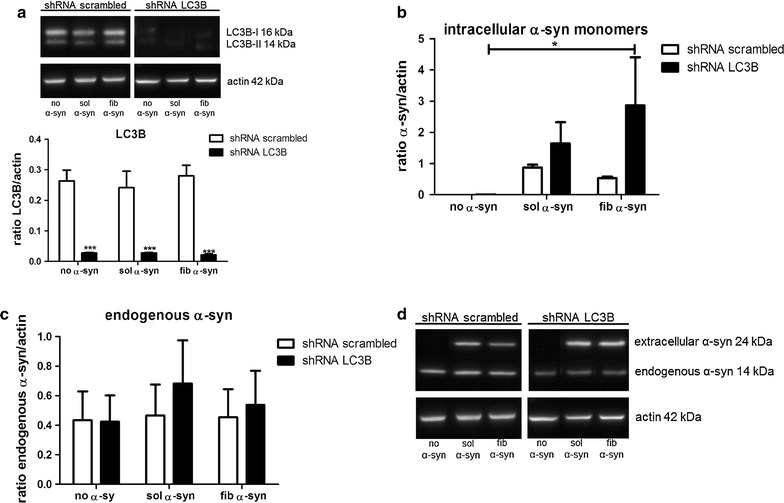
Genetic LC3B knockdown does not induce GCI-like formation of extracellularly incorporated recombinant sol and fib α-syn species in oligodendroglial cells. Oligodendroglial cells were transfected using the shRNA plasmid against LC3B ligated to GFP to create a constitutive knockdown of LC3B in oligodendroglial cells. Western blot analysis confirmed a highly significant down-regulation of the LC3B protein in the transfected oligodendroglial cultures irrespective of the treatment with sol and fib α-syn. LC3B levels were normalized to actin levels and a control lysate (**a**). Intracellular amounts of uptaken α-syn monomers and endogenous α-syn were measured in oligodendroglial cells exposed to extracellular sol or fib α-syn combined with LC3B knockdown using Western blot analysis. α-Syn levels were normalized to actin levels. Minor incorporation of sol and fib α-syn was found in oligodendroglial cells upon transfection with the control plasmids (scrambled shRNA). Knockdown of LC3B revealed an increased uptake of sol and fib α-syn compared to untreated cells and to cells transfected with the scrambled shRNA. However, only treatment with fib α-syn induced a significantly increased incorporation of α-syn upon LC3B knockdown compared to untreated cells. No significant difference was detected comparing LC3B knockdown and scrambled shRNA transfected oligodendroglial cells (**b**, **d**). No differences between α-syn levels were found regarding any treatment when measuring endogenously expressed α-syn in oligodendroglial cells (**c**, **d**). Two-way analysis of variance with post hoc Bonferroni test was applied. Data are presented as mean ± SEM. *p < 0.05; ***p < 0.001. N number equals 4

The genetic knockdown was verified using Western blot analysis revealing a significant reduction of the LC3B protein in oligodendroglial cells transfected with the shRNA against LC3B compared to the scrambled shRNA plasmid (Fig. 7a).

LC3B knockdown in oligodendroglial cells did not reveal a significant effect regarding the levels of incorporated extracellular added α-syn compared to oligodendroglia transfected with a control plasmid. No oligomers and fibrils were detectable. Treatment with sol and fib α-syn of oligodendroglia transfected with the scrambled shRNA plasmid presented with uptake of α-syn, yet the incorporation was not significant. In contrast, LC3B knockdown induced an increased incorporation of α-syn compared to untreated oligodendroglial cells, but only the intracytoplamatic accumulation of fib α-syn was significantly enhanced. Yet, α-syn levels in LC3B knockdown oligodendroglial cells did not reach a significant increase compared to cells transfected with the control plasmid upon α-syn challenge. No oligomers and fibrils were detectable (Fig. 7b, d). Moreover, low amounts of endogenously expressed α-syn were detectable in all shRNA transfected oligodendroglial cells. Knockdown of the LC3B gene did not change levels of endogenous α-syn significantly in oligodendroglia compared to control cells (Fig. 7c, d).

**Fig. 8:**
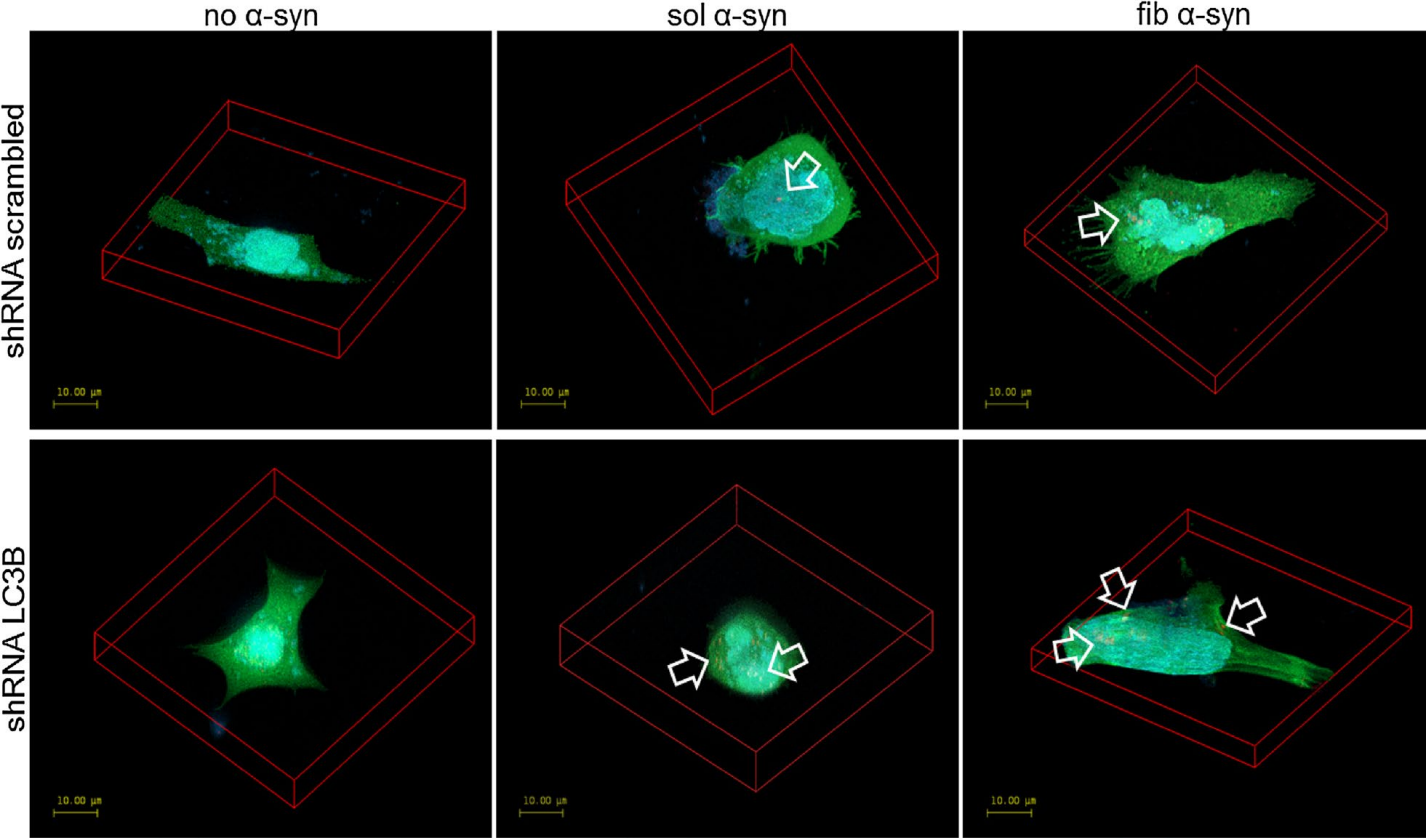
Morphological analysis reveals small α-syn-positive inclusions however no GCI-like formation upon LC3B knockdown in oligodendroglial cells. Confocal microscopy was performed to analyze the incorporation and inclusion formation of sol and fib α-syn in oligodendroglial cells upon genetic knockdown of LC3B. Immunocytochemistry was accomplished following fixation labelling human α-syn (15G7, red), GFP ligated to the shRNA plasmid (transfection control, green) and the nucleus using DAPI (blue). α-Syn-positive inclusion were found in oligodendroglia transfected with shRNA scrambled and shRNA LC3B. No differences were detected regarding the incorporation of α-syn comparing the treatment with sol and fib α-syn. However, increased number of smaller α-syn-positive dots was detected in LC3B knockdown compared with control transfected oligodendroglia. Yet, no GCI-like accumulation was found in any of the treated cells

We observed that oligodendroglia transfected with the shRNA plasmid against LC3B were able to incorporate sol and fib α-syn and compared to cells transfected with the scrambled shRNA more punctuate α-syn inclusions were found. Yet, no differences regarding incorporated sol and fib α-syn were found during morphological analyses at the confocal microscope. Furthermore, no GCI formation was observed in any of the analyzed oligodendroglial cells with LC3B knockdown (Fig. 8).

## Discussion

The aggregation of α-syn in oligodendroglial cells, so called GCIs, is the major pathological hallmark of MSA. A connection between the formation of these α-syn-positive inclusions and neuronal degeneration and disease progression respectively has been postulated [31, 53]. However, to date the formation of GCIs in MSA has not been elucidated. Different mechanisms, including autophagy or proteasome dysfunction, are thought to be involved in the development of α-syn-positive inclusions in oligodendroglial cells [11, 32]. In the current study, we investigated the impact of dysfunctional macroautophagy on exposure to extracellular α-syn and formation of α-syn-positive inclusions in oligodendroglia. We observed no GCI-like formation upon pharmacological blocking of the fusion of the autophagosome and the lysosome, as well as genetic knockdown of LC3B combined with the treatment with recombinant extracellular sol and fib α-syn species.

Incorporation of extracellular or neuronal derived α-syn by oligodendroglial cells has already been shown in various studies in vitro and in vivo [22, 24–26]. In our cell culture model, we were able to demonstrate the uptake of extracellularly added recombinant sol and fib α-syn. Furthermore, we provide evidence that the incorporation of α-syn and the formation of inclusions by MO 3.13 oligodendroglia is comparable to murine primary oligodendroglial cells suggesting that the oligodendroglial cell line is a reliable tool for further experiments. Autophagy mechanisms have been considered as important and efficient α-syn clearance mechanisms [27–29, 41, 43, 44]. An involvement of autophagy pathways regarding α-syn accumulation in oligodendroglial cells in MSA or other α-synucleinopathies seems conclusive [11, 45]. Furthermore, it was shown that extracellularly added α-syn and the overexpression of α-syn alone did not inhibit the autophagic flux in oligodendroglia as suggested by unchanged LC3-II levels among others [24]. Similar results were found in our experiments. The addition of extracellular recombinant sol and fib α-syn did not induce an increase in LC3B-II levels indicating a normal autophagic flux as described previously. Moreover, blocking of macroautophagy with BAF is known to inhibit the degradation of LC3B-II suggesting a successful block of the fusion of the autophagosome with the lysosome in the treated cells [50, 51]. In our experiments, we confirm a successful inhibition of macroautophagy by adding BAF to oligodendroglial cells irrespective of the treatment with sol or fib α-syn, showing significantly increased LC3B-II levels.

Most cells control their need for energy through autophagy, and oxidative stress or the presence of ROS might interfere with the autophagy pathways as ROS have been associated with an activated autophagy upon nutrient deprivation, yet the connection between oxidative stress and autophagy is far from being elucidated [54]. Furthermore, damage to mitochondria was shown to impair the autophagic flux and lead to α-syn accumulation in oligodendroglial cells [24]. We found that the block of macroautophagy through BAF induces ROS production in oligodendroglia, which can be enhanced by the addition of fib α-syn. Yet, extracellularly added α-syn alone does not induce ROS production in oligodendroglia suggesting an important role of inhibited autophagy in the oxidative stress pathway. In accordance with our data, it was already shown that autophagy deficiency can induce oxidative stress and mitochondrial ROS production [55]. However, although we successfully blocked macroautophagy and thus induced oxidative stress in oligodendroglia, we were not able to detect an accumulation of α-syn and ubiquitin relevant to GCI-like formation in our cell culture model upon treatment with extracellular added α-syn. Analyses at the confocal microscope revealed smaller α-syn inclusions but we did not detect any α-syn aggregates in oligodendroglia treated with recombinant α-syn, as well as they did not resemble GCI-like inclusions as described in MSA brains [56]. In a recent study, the inhibition of macroautophagy with ammonium chloride and chloroquine as well as induced oxidative stress in OLN-t40 oligodendroglia led to the accumulation of α-syn in the cytoplasm [24], yet GCI-like inclusions were also not reported. Only small amounts of incorporated α-syn were found in oligodendroglial cells untreated or treated with BAF. However, we did not induce mitochondrial impairment leading to a halt in the autophagic flux in our model suggesting that macroautophagy blocking through BAF is not enough to induce GCI-like formation in oligodendroglial cells. Probably more than one pathway dysfunction and in particular a combination of factors is needed to induce GCI formation in oligodendroglial cells and mimic GCIs as seen in MSA. Another limitation of this study could be the difference regarding oligodendroglial features and also GCI formation properties comparing oligodendroglial cells in vivo and in vitro. Macroautophagy dysfunction in oligodendroglia in vivo could reveal a different outcome regarding α-syn accumulation compared to treated oligodendroglial cell lines. Furthermore, it can also be discussed that the results in various studies depend on the oligodendroglial cell type used in the experiments and therefore the differing results could indicate different properties of the oligodendroglial cell lines and primary oligodendroglial cells used. Moreover, a 24 h-duration of α-syn treatment and the concentration of 18 µg/mL added, might be not enough for oligodendroglia to build up larger α-syn-positive inclusion, as the incorporation of α-syn has been stated to be time- and concentration-dependent [25, 26]. In a different study, it is suggested that BAF not only potentiates the toxicity of aggregated α-syn species, but also induces a reduction of the α-syn aggregation and furthermore, the secretion of toxic α-syn species by neuronal cells into the extracellular space [42, 47]. This could be an explanation for the low levels of α-syn upon BAF treatment in oligodendroglial cells in our study. However, in future experiments α-syn release would need to be analyzed to support this hypothesis.

In a next step, the genetic knockdown of LC3B was performed in oligodendroglial cells followed by treatment with recombinant sol and fib α-syn. We observed incorporation of α-syn in oligodendroglial cells lacking LC3B compared to cells treated with the control/scrambled shRNA similar to a previously published experiment showing that the down-regulation of Atg5 in oligodendroglial cells leads to an increase of α-syn levels [24]. Furthermore, confocal analysis of the cells revealed more dots of incorporated α-syn in oligodendroglia with reduced LC3B levels suggesting that a genetic knockdown of the macroautophagy gene LC3B might induce accumulation of α-syn. However, no GCI-like formation was observed. As mentioned above, higher α-syn concentration or longer incubation times have to be tested in further experiments. However, the release of α-syn through exosomes by oligodendroglia triggered by macroautophagy dysfunction as described in neurons [42, 47] might be another explanation regarding the lack of GCI-like inclusions.

## Conclusion

In conclusion, in this study we demonstrate that blocking of macroautophagy in the presence of sol or fib α-syn does not lead to the formation of α-syn inclusions in oligodendroglia as observed in human MSA. However, we found that macroautophagy blocking leads to higher oxidative stress in combination with fib α-syn treatment. Further studies have to be conducted to clarify the role of macroautophagy in the initiation and progression of MSA. Defects in different pathways might contribute to the formation of MSA-like GCIs. The investigation of the CMA pathway and its role in GCI formation in oligodendroglia could be another interesting next step in future studies, as in neuronal cells CMA is shown to have a leading role in α-syn degradation [43]. Furthermore, it is described that macroautophagy dysfunction induces the release of α-syn through exosomes by neuronal cells [42, 47]. The same mechanism might be also true for oligodendroglial cells, which could be an explanation that macroautophagy blocking does not induce GCI-like formation in our experiments. In summary, our results suggest that macroautophagy dysfunction is not the only pathway involved in the formation process of GCIs in MSA, suggesting a combination of different approaches (e.g. proteasome and autophagy inhibition) in future studies. Furthermore, in-depth studies are required to transfer the relevance of the current findings to the situation in patients.

## Abbreviations

α-syn: α-synuclein
BAF: bafilomycin A1
bFGF: basic fibroblast growth factor
CMA: chaperone mediated autophagy
CNTF: ciliary neurotrophic factor
DAPI: 4′,6-diamidino-2-phenylindole dihydrochloride
DLB: dementia with Lewy bodies
DMEM: Dulbecco’s modified Eagle’s medium
FCS: fetal calf serum
fib α-syn: fibrillar α-synuclein
GCIs: glial cytoplasmic inclusions
GFP: green fluorescent protein
kDa: kilodalton
LAL: limulus amoebocyte lysate
LBs: Lewy bodies
LC3B: microtubule-associated protein 1 light chain 3B
MSA: multiple system atrophy
NAC: N-acetyl-l-cysteine
NBT: nitroblue tetrazolium chloride
OPC: oligodendrocyte precursor cell
PBS: phosphate buffered saline
PBS-T: phosphate buffered saline containing 0.1% Tween-20
PDGF-AA: platelet-derived growth factor AA
PDL: poly-d-lysine
PFA: paraformaldehyde
RT: room temperature
sol α-syn: soluble α-synuclein
TIT: triiodothyronine
UPS: ubiquitin–proteasome system
V-ATPase: vacuolar H + ATPase
WGA: wheat germ agglutinin
